# A 3D-Printed Pump-Free Multi-Organ-on-a-Chip Platform for Modeling the Intestine–Liver–Muscle Axis

**DOI:** 10.3390/mi17020180

**Published:** 2026-01-28

**Authors:** Rodi Kado Abdalkader, Takuya Fujita

**Affiliations:** 1 Ritsumeikan Global Innovation Research Organization (R-GIRO), Ritsumeikan University, Kusatsu 525-8577, Shiga, Japan; fujita-t@ph.ritsumei.ac.jp; 2 Department of Pharmaceutical Sciences, Ritsumeikan University, Kusatsu 525-8577, Shiga, Japan

**Keywords:** multi-organ-on-a-chip, intestine–liver–muscle axis, pump-free, 3D printing

## Abstract

The intestine–liver–muscle axis plays an essential role in drug and nutrient absorption, metabolism, and energy balance. Yet in vitro models capable of recapitulating this inter-organ communication remain limited. Here, we present a pump-free, 3D-printed multi-organ-on-a-chip device that enables dynamic co-culture of Caco-2 intestinal epithelial cells, HepG2 hepatocytes, and primary human skeletal myoblasts (HSkMs) under gravity-driven oscillatory flow. The device consists of five interconnected chambers designed to accommodate Transwell cell culture inserts for intestine and muscle compartments and hydrogel-embedded hepatocyte spheroids in the central hepatic compartment. The device was fabricated by low-cost fused deposition modeling (FDM) using acrylonitrile butadiene styrene (ABS) polymers. Under dynamic rocking, oscillatory perfusion promoted inter-organ communication without the need for external pumps or complex tubing. Biological assessments revealed that dynamic co-culture significantly enhanced the characteristics of skeletal muscle, as indicated by increased myosin heavy chain expression and elevated lactate production, while HepG2 spheroids exhibited improved hepatic function with higher albumin expression compared with monoculture. Additionally, Caco-2 cells maintained stable tight junctions and transepithelial electrical resistance, demonstrating preserved intestinal barrier integrity under dynamic flow. These results establish the device as a versatile, accessible 3D-printed platform for modeling the intestine–liver–muscle axis and investigating metabolic cross-talk in drug discovery and disease modeling.

## 1. Introduction

The human body maintains metabolic homeostasis through tightly coordinated interactions among multiple organ systems. Among these, the intestine, liver, and skeletal muscle form a central axis that governs nutrient absorption, energy metabolism, and the pharmacokinetics of orally administered drugs [1,2,3]. The intestine acts as the initial barrier for nutrients and xenobiotics, controlling absorption through its polarized epithelial lining and selectively regulating transport into the portal circulation [4]. Following absorption, the liver serves as the primary site for first-pass metabolism, performing a wide range of enzymatic transformations that determine drug bioavailability and systemic exposure [5,6]. The skeletal muscle, as the largest metabolic organ, is a major site of glucose and fatty acid utilization, responding to circulating metabolites and hormonal cues to maintain systemic energy balance [7]. Disruption within this axis underlies a spectrum of clinical conditions, including insulin resistance, sarcopenia, and non-alcoholic fatty liver disease, highlighting the importance of accurately modeling inter-organ communication for both basic research and drug development [8,9].

Conventional in vitro models, such as static monocultures or simple co-cultures, are limited in their ability to replicate the dynamic exchange of nutrients, hormones, and metabolites between organs [10]. Transwell cell culture systems, where cells are cultured on semi-permeable membranes, provide a partial solution by mimicking tissue barriers and allowing selective transport, and they are widely used for evaluating intestinal permeability and epithelial function [11]. However, traditional Transwell-based approaches remain static and fail to reproduce the dynamic fluidic conditions and multi-organ interactions that occur in vivo [12]. Meanwhile, in vivo studies in animal models offer insights into systemic metabolism but are constrained by interspecies differences in transporter expression, enzyme activity, and tissue response, which often limit the predictive value of preclinical data for human outcomes [13,14].

Organ-on-a-chip (OoC) and microphysiological systems (MPSs) have emerged as transformative platforms to bridge this gap, enabling the recapitulation of human tissue function under controlled microfluidic environments [15,16]. By integrating multiple tissue types into interconnected compartments, OoC systems allow the study of organ-to-organ communication, drug absorption, and metabolism with higher physiological relevance [17]. However, many existing OoC platforms rely on external peristaltic pumps or complex microfluidic controllers, which increase operational complexity, cost, and the technical barrier for routine use in standard laboratories. To overcome these limitations, we used our previously developed platforms [18,19] as the basis for a 3D-printed, pump-free multi-organ-on-a-chip device that integrates Transwell-based culture insert compartments for intestine, liver, and skeletal muscle. The use of 3D printing enables rapid prototyping, low-cost production, and customizable design, making the platform accessible for widespread implementation. Moreover, the gravity-driven bidirectional flow generated by simple rocking eliminates the need for external pumps while maintaining physiologically relevant inter-organ exchange.

In this study, we present a gravity-driven, 3D-printed multi-organ-on-a-chip platform that models the intestine–liver–muscle axis. Caco-2 cells on Transwells cell culture inserts formed an intestinal barrier, human skeletal myoblasts provided a metabolically active muscle compartment, and HepG2 spheroids embedded in collagen/Matrigel served as the liver component for first-pass metabolism. The device, fabricated by low-cost 3D printing using acrylonitrile butadiene styrene (ABS) polymer with PDMS sealing, operates under gentle rocking to generate pump-free, bidirectional flow. By integrating Transwell cell culture inserts with dynamic microfluidics, the system offers a scalable and accessible platform for physiologically relevant co-culture for the study of inter-organ communication.

## 2. Methods

### 2.1. Device Fabrication and Characterization

The multi-organ-on-a-chip device was fabricated following our previously reported method [18,19] using fused deposition modeling (FDM) 3D printing (Shenzhen Anycubic Technology Co., Ltd., Shenzhen, China) with 1.75 mm acrylonitrile butadiene styrene (ABS) filaments. Computer-aided design (CAD) models were generated using Tinkercad (https://www.tinkercad.com) (Supplementary Figure S1), and STL files were processed using Ultimaker Cura 5.11 software. Printing parameters were set to a layer height of 0.01–0.02 mm and a shell thickness of 2–10 layers. Printed devices were adhered to PET clear dishes using PDMS, while Polylactic acid (PLA) lattice inserts were printed separately to support hydrogel encapsulation. After printing, excess edges were trimmed, and devices were stored at room temperature until use. To assess fluid dynamics within the microchannels, fluorescein sodium (1 mg mL^−1^, Nacalai Tesque (Kyoto, Japan)) was perfused under rocking conditions, and oscillatory flow was confirmed using bright-field microscopy. To support the experimental flow visualization, a two-dimensional simulation of oscillatory fluid motion in the circulation channel (40 mm × 4 mm× 2 mm; L × W × H) was performed. The flow was modeled as laminar with a parabolic velocity profile, and the mean velocity was set to oscillate sinusoidally with time to mimic platform rocking (±6°, 10 cycles min^−1^, peak velocity ≈ 5 mm s^−1^ as measured experimentally). Tracer transport was described by the advection–diffusion equation using the diffusion coefficient of fluorescein in water (4 × 10^−10^ m^2^ s^−1^) [20]. The equation was solved numerically with a finite-difference method in Python 3.11 (NumPy, Matplotlib). No-flux boundary conditions were applied at the channel walls. Simulation output was rendered as animations showing tracer oscillation and mixing (Supplementary Code S1).

### 2.2. Cell Culture and Multi-Organ Assembly

Caco-2 cells (RIKEN BioResource Research Center (BRC), Tsukuba, Ibaraki, Japan) were maintained in DMEM (Wako, Osaka, Japan) supplemented with 10% fetal bovine serum (FBS; Thermo Fisher Scientific, Waltham, MA, USA), 1% non-essential amino acids (Nacalai Tesque), and 1% penicillin–streptomycin (Wako). Cells were seeded at 0.5 × 10^5^ cells per insert onto 24-well polyester Transwell inserts (0.4 µm pore size, ThinCert; Greiner Bio-One GmbH, Kremsmünster, Austria), with medium supplied apically (0.25 mL) and basolaterally (0.75 mL). Medium was exchanged every other day. After 14 days, Caco-2 cells formed polarized epithelial monolayers, confirmed by stable transepithelial electrical resistance (TEER ≥ 200 Ω·cm^2^).

Human primary normal skeletal myoblasts (HSkM; Gibco, Thermo Fisher Scientific, Waltham, MA, USA) were cultured in DMEM (Wako) supplemented with 2% horse serum (Thermo Fisher Scientific) to induce differentiation. Cells were seeded at a density of 2.4 × 10^4^ per Transwell insert and incubated for 24 h at 37 °C with 5% CO_2_. The culture medium was replaced daily with fresh differentiation medium. After 2 days, confluent monolayers were obtained and used for device integration.

HepG2 cells (RIKEN BRC) were suspended in a collagen I/Matrigel mixture (Cellmatrix I-A, Nitta Gelatin Inc. (Osaka, Japan)/Geltrex, Thermo Fisher Scientific) at a 9:1 ratio (final collagen concentration 3 mg mL^−1^) at a density of 4 × 10^5^ cells mL^−1^. Droplets (125 µL) of the suspension were dispensed into PLA lattice inserts placed in 12-well plates and allowed to gel at 37 °C for 30 min. Constructs were cultured in DMEM supplemented with 10% FBS, 1% non-essential amino acids, and 1% penicillin–streptomycin for 5 days, during which HepG2 cells formed compact spheroids within the hydrogel scaffold.

At day 0 of co-culture, devices were sterilized by UV irradiation for 60 min, and preconditioned Caco-2 monolayers (14 days), HSkM monolayers (2 days), and HepG2 spheroids (5 days) were integrated. Transwell inserts containing Caco-2 and HSkM were positioned in the designated side chambers, while HepG2 hydrogel constructs were placed in the central hepatic compartment. The assembled devices were mounted on a rocking platform (±6°, 10 cycles min^−1^) to generate gravity-driven bidirectional flow through the interconnecting circulation channel or maintained under static conditions. The circulation channel was filled with shared DMEM supplemented with 10% FBS, 1% non-essential amino acids, and 1% penicillin–streptomycin, while the apical compartments of Caco-2 and HSkM inserts were maintained with their respective culture media. All media were replenished every 24 h.

### 2.3. Functional Assays

Barrier integrity of Caco-2 monolayers was assessed by transepithelial electrical resistance (TEER) using an EVOM2 voltohmmeter (World Precision Instruments (Sarasota, FL, USA)) with chopstick electrodes. Skeletal muscle metabolic activity was evaluated by measuring lactate concentration in the circulation channel using a colorimetric lactate assay (Dojindo Laboratories (Kumamoto, Japan)) according to the manufacturer’s instructions.

For immunofluorescence (IF) assays, cells and spheroids were fixed with 4% paraformaldehyde for 20 min at room temperature, permeabilized with 0.1% Triton X-100 in PBS, and blocked with 5% bovine serum albumin (BSA) in PBS for 1 h. Samples were incubated overnight at 4 °C with the following primary antibodies: ZO-1 (rabbit anti-human; Proteintech Group, Inc. (Rosemont, IL, USA)) for tight junctions, albumin (ALB, mouse anti-human; Bioss Inc. (Woburn, MA, USA)) for hepatocytes, and myosin heavy chain (MYH, clone B-5, Alexa Fluor 594 conjugate; Thermo Fisher) for skeletal muscle. After washing, samples were incubated for 1 h at room temperature with species-appropriate secondary antibodies, including Alexa Fluor 488 goat anti-rabbit and Alexa Fluor 555 goat anti-mouse (Thermo Fisher Scientific). Nuclei were counterstained with DAPI (Thermo Fisher Scientific) before imaging. Imaging was performed using fluorescence microscopy (KEYENCE, Tokyo, Japan).

### 2.4. Data Analysis and Statistics

For the investigation of cell morphology and quantification of corrected total fluorescence intensity (CTCF), images were analyzed using ImageJ software 1.53k (National Institutes of Health, Bethesda, MD, USA) and CellProfiler software [21] (Version 3.1.8; Broad Institute of Harvard and MIT, Cambridge, MA, USA). All quantitative data are presented as mean ± standard error of the mean (SEM). Statistical analyses were performed using one-way ANOVA with Dunnett’s multiple comparison test or unpaired two-tailed Student’s *t*-test, as appropriate. Graphs and statistical outputs were generated using GraphPad Prism 9 (GraphPad Software, San Diego, CA, USA).

## 3. Results

### 3.1. Design and Fabrication of a Multi-Organ-on-a-Chip Device

**Figure 1:**
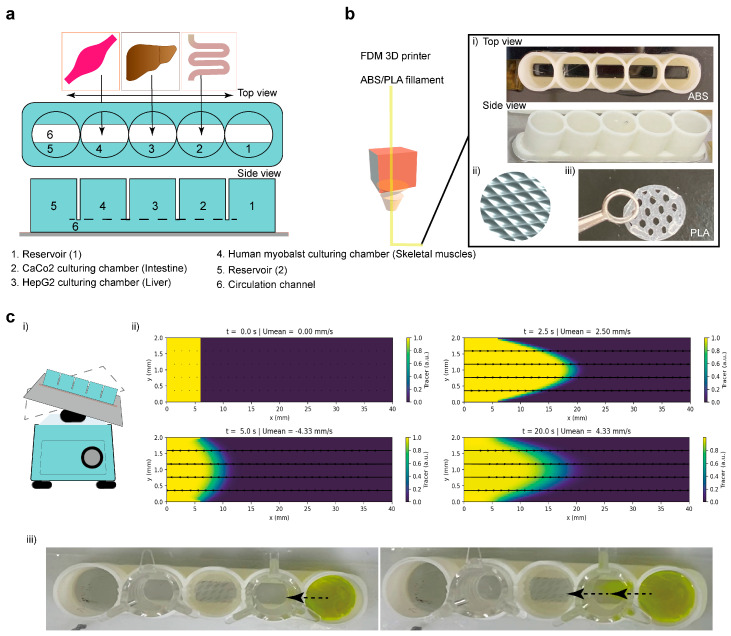
Design and fabrication of a 3D printed multi-organ-on-a-chip device for gravity-driven co-culture of intestine, liver, and skeletal muscle tissues. (**a**) Schematic of the device showing the arrangement of organ specific chambers and reservoirs: (1) inlet reservoir, (2) Caco-2 intestinal chamber, (3) HepG2 hepatic chamber, (4) HSkM human skeletal myoblast chamber, (5) outlet reservoir, and (6) connecting circulation channel. Arrows indicate the direction of gravity-driven bidirectional flow during rocking. (**b**) Fabrication of the device using fused deposition modeling (FDM) 3D printing. (i) Photograph of the printed ABS body with five aligned organ chambers. (ii) CAD illustrating the internal lattice used to support hydrogel or ECM seeding. (iii) PLA lattice insert for hydrogel embedding and enhanced cell attachment. (**c**) Dynamic perfusion setup and demonstration of oscillatory flow. (i) Device mounted on a rocking platform (±6°) to generate laminar bidirectional flow without external pumps. (ii) Rocking-flow simulation showing tracer transport in a 40 mm length × 2 mm height. Snapshots at indicated times display tracer concentration (heatmap) and instantaneous axial velocity (arrows) at 0 s, 2.5 s, 5 s, 7.5 s and 20 s generated by sinusoidal rocking at 10 cpm. The model solves the advection–diffusion equation with a Poiseuille flow profile. (iii) Experimental observation of tracer (fluorescein sodium) oscillating through the interconnected chambers, confirming passive mixing and nutrient transport across the multi-organ system. Arrows indicate the direction of gravity-driven bidirectional flow under rocking. Artwork created by the corresponding author (Rodi Kado Abdalkader).

We first developed a 3D-printed multi-organ-on-a-chip device to enable gravity-driven co-culture of intestine, liver, and skeletal muscle tissues without the use of external pumps (Figure 1a). The device comprised five aligned circular chambers: two reservoirs at the ends, one central chamber for HepG2 hepatocytes, and two side chambers for Caco-2 intestinal and HSkM human skeletal myoblast cultures, all interconnected by a lower circulation channel. Arrows in the schematic indicate the bidirectional flow path generated by gentle rocking of the device at ±6°, which drives fluid oscillation and nutrient exchange between compartments.

The device body was fabricated using FDM 3D printing with ABS for the main structure and PLA inserts for hydrogel support (Figure 1b). The modular design included a lattice structure to secure collagen/Matrigel droplets for hepatocyte spheroid culture, and flat surfaces compatible with Transwell cell culture inserts for the intestinal and muscle compartments. Figure 1c(ii) shows a time-lapse representation of the simulated dye gradient profile within the microchannel, illustrating oscillatory medium movement generated by the rocker system. The alternating flow direction (±6° tilt, 10 cycles/min) allowed repeated fluid exchange between chambers. The experimental dye visualization confirmed the predicted oscillatory pattern and supports effective inter-chamber communication (Figure 1c, Supplementary Figure S1 and Video S1). Consistent with the device geometry and recorded mean velocity, the circulation channel exhibited laminar flow (Re ≈ 14.8) with an estimated volumetric flow rate of 44.4 µL·s^−1^. The corresponding wall shear stress was calculated as 0.167 dyn·cm^−2^ (Supplementary Information). The pressure drop across a 5.55 mm path was approximately 0.0925 Pa, confirming stable, low-resistance perfusion. Overall, these parameters place the device within a gentle-shear, laminar regime (≈0.01–0.02 Pa; 0.1–0.2 dyn·cm^−2^) that aligns well with physiological shear levels reported for intestinal and hepatic microphysiological systems [22,23]. The observed back-and-forth dye oscillation further demonstrated that the gravity-driven flow was sufficient to perfuse all chambers, enabling continuous inter-organ communication without the need for external pumps or complex microfluidic systems.

### 3.2. Cell Preparation and Integration into the Device

**Figure 2:**
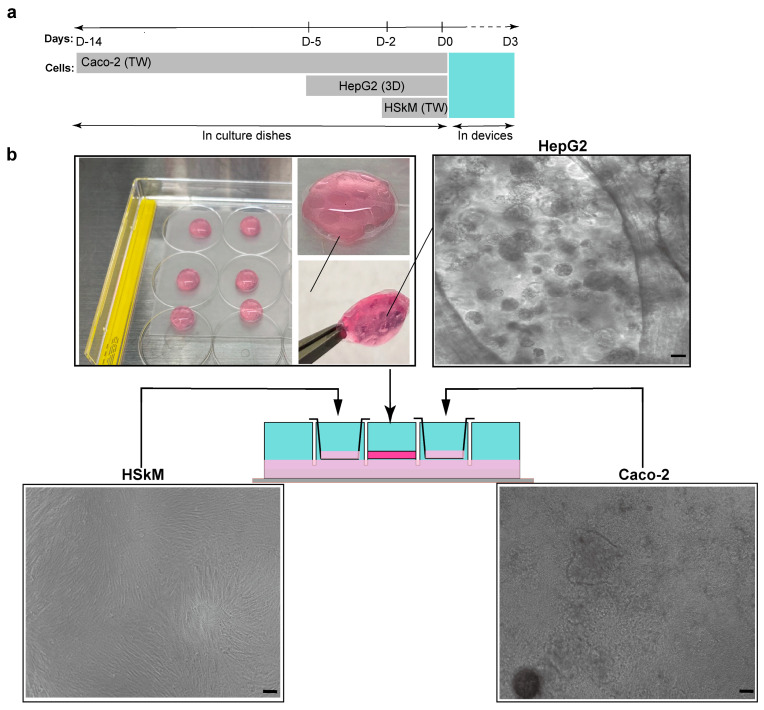
Cell preparation and integration workflow for multi-organ co-culture in the 3D-printed device. (**a**) Timeline of cell preparation and device assembly. Caco-2 cells were pre-cultured in Transwell cell culture inserts for 14 days to form polarized intestinal monolayers, while HepG2 hepatocytes were aggregated into 3D spheroids over 5 days in collagen/Matrigel droplets, and HSkM primary human skeletal myoblasts were pre-cultured for 2 days in Transwells. At day 0 (D0), the three tissue types were integrated into the device for 3 days of dynamic co-culture under gravity-driven bidirectional flow. (**b**) Representative images of individual tissue compartments prior to integration: HSkM (**left**): Confluent myoblast monolayer after 2 days of culture. HepG2 (**center**): Collagen/Matrigel spheroids after 5 days of culture, shown as whole droplets (inset) and under brightfield microscopy. Caco-2 (**right**): Polarized monolayer in Transwell cell culture insert after 14 days. Scale bars, 50 µm. The schematic below illustrates the spatial arrangement of the three tissues in the multi-organ-on-a-chip device, with Transwell cell culture inserts for intestinal and muscle compartments and a central hydrogel chamber for hepatic spheroids.

To establish a physiologically relevant multi-organ system, we optimized the timing and configuration of cell preparation prior to integration (Figure 2a). Caco-2 cells were pre-cultured in Transwell cell culture inserts for 14 days to form polarized monolayers. HepG2 cells were encapsulated in collagen/Matrigel to generate 3D spheroids over 5 days, providing a liver-like 3D microenvironment. HSkM cells were pre-cultured for 2 days to establish a confluent monolayer in Transwells before assembly into the chip. At day 0, all three tissues were transferred to the device, where they were co-cultured under gravity-driven oscillatory flow for 3 days to enable inter-organ communication. Representative images show the morphology and maturity of each tissue prior to device integration (Figure 2b). HSkM monolayers exhibited elongated, confluent morphology after 2 days, while HepG2 cells formed compact 3D spheroids within hydrogel droplets by day 5. Caco-2 monolayers displayed a uniform epithelial layer consistent with functional barrier formation after 14 days. The schematic illustrates show these preconditioned tissues were spatially arranged in the device, with intestinal and muscle compartments in Transwell cell culture inserts and the hepatic compartment embedded in the central hydrogel chamber. This preparation strategy allowed all three tissues to be introduced in a functionally ready state, supporting immediate cross-talk upon initiation of dynamic culture.

### 3.3. Dynamic Co-Culture Enhances Skeletal Muscle Maturation and Metabolic Activity

**Figure 3:**
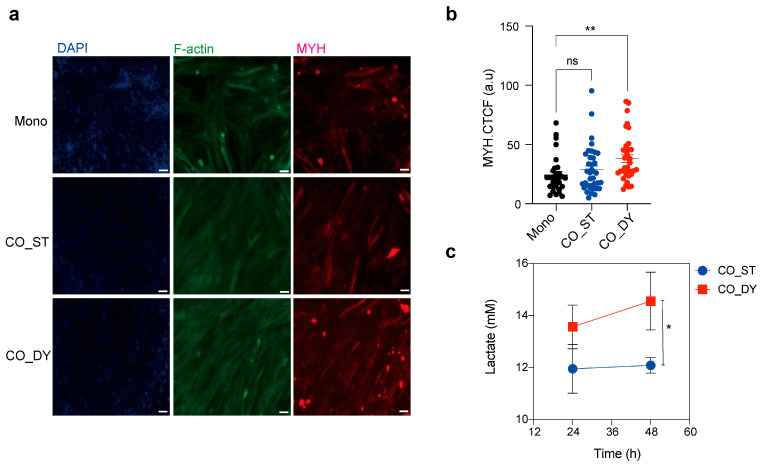
Skeletal muscle differentiation and metabolic activity under mono- and co-culture conditions. (**a**) Immunofluorescence staining of human skeletal myoblasts cultured in monoculture (Mono), in the multi-organ device under static conditions (CO_ST), and in the device under dynamic rocking conditions (CO_DY) at day 3. Nuclei (DAPI, blue), F-actin (green), and myosin heavy chain (MYH, red) are shown. Scale bars, 50 µm. (**b**) Quantification of MYH expression by corrected total cell fluorescence (CTCF) in Mono, CO_ST, and CO_DY cultures. Dynamic co-culture significantly enhanced MYH expression compared with static and monoculture conditions (one-way ANOVA with Dunnett’s post hoc test, ** *p* < 0.01; ns = not significant). (**c**) Lactate concentration measured in the circulation channel under static (ST) and dynamic (DY) co-culture conditions at 24–48 h. Dynamic culture resulted in significantly higher lactate release compared with static conditions (unpaired Student’s *t*-test, * *p* < 0.05). Data are presented as mean ± SEM from three independent biological replicates (*n* = 3).

To evaluate skeletal muscle differentiation, HSkM cells were cultured as monocultures (Mono) or co-cultured in the device under static (CO_ST) or dynamic (CO_DY) conditions. Immunofluorescence staining for myosin heavy chain (MYH) revealed more extensive myotube formation in co-culture compared to monoculture, with the strongest expression observed under dynamic conditions (Figure 3a). Quantitative analysis of MYH expression confirmed a significant increase in CO_DY compared with Mono and CO_ST cultures (Figure 3b). In parallel, lactate levels measured in the circulation channel were significantly higher in CO_DY compared to CO_ST, indicating enhanced glycolytic activity under dynamic flow (Figure 3c). Together, these results demonstrate that dynamic co-culture promotes both maturation and metabolic activity of HSkM.

### 3.4. Albumin Expression in HepG2 Spheroids Is Enhanced Under Dynamic Co-Culture

**Figure 4:**
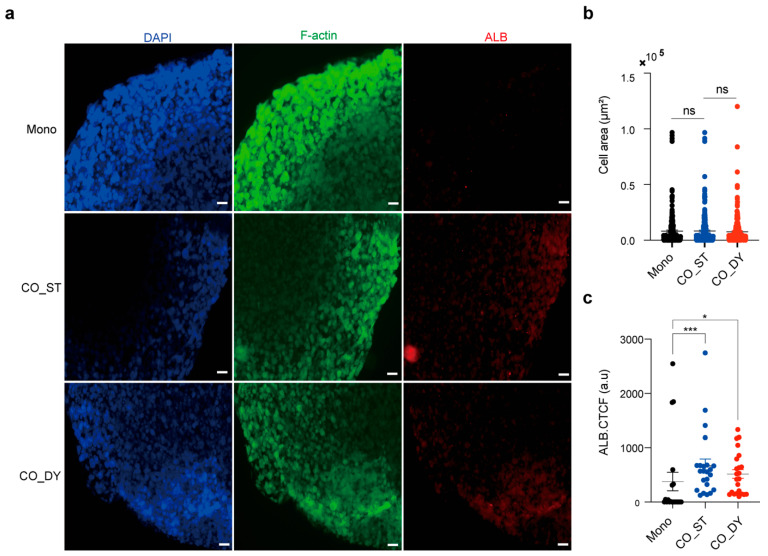
Morphology and functional activity of HepG2 cells under mono- and co-culture conditions. (**a**) Immunofluorescence staining of HepG2 spheroids showing nuclei (DAPI, blue), F-actin (green), and albumin (ALB, red). Albumin expression was markedly enhanced under co-culture conditions, with the strongest expression observed under dynamic flow. Scale bars, 200 µm. (**b**) Quantification of cell aggregates area under monoculture (Mono), multi-organ device under static (CO_ST), and multi-organ device dynamic rocking (CO_DY) conditions, showing no significant change among groups (ns = not significant). (**c**) Quantification of ALB expression by corrected total cell fluorescence (CTCF) in Mono, CO_ST, and CO_DY conditions. Both static and dynamic co-cultures significantly increased ALB expression compared with monoculture (one-way ANOVA with Dunnett’s post hoc test; *** *p* < 0.001, * *p* < 0.05). Data are presented as mean ± SEM from three independent biological replicates (*n* = 3).

HepG2 cells encapsulated in collagen/Matrigel scaffolds maintained compact spheroid morphology with no significant differences in spheroid areas across all conditions (Figure 4a,b). In contrast, immunofluorescence staining revealed stronger albumin (ALB) expression in co-culture groups compared with monocultures. Quantitative analysis confirmed that both static co-culture and dynamic co-culture significantly enhanced ALB intensity relative to monoculture (Figure 4c). Notably, dynamic co-culture showed a further increase in ALB expression compared with static co-culture, although this difference did not reach statistical significance. It should be noted that HepG2 cells are a convenient and reproducible hepatic model for proof-of-concept studies but exhibit limited drug-metabolizing capacity, including relatively low cytochrome P450 activity. Accordingly, the ALB readout here is interpreted as an indicator of improved hepatic phenotype/maintenance within the platform rather than a quantitative demonstration of CYP-mediated drug metabolism. Together, these findings indicate that co-culture with other tissues in the device supports improved hepatic functional markers in HepG2 spheroids.

### 3.5. Caco-2 Intestinal Barrier Integrity Is Preserved Under Dynamic Conditions

**Figure 5:**
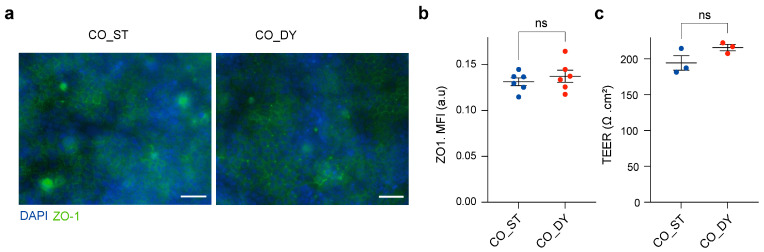
Barrier integrity of Caco-2 monolayers under static and dynamic co-culture conditions. (**a**) Immunofluorescence staining of Caco-2 cells for tight junction; ZO-1 (green) and nuclei (DAPI, blue) in the multi-organ device under static (CO_ST) and dynamic rocking (CO_DY) conditions. Large blue frame-like features (>100 µm) in the DAPI channel arise from the Transwell membrane/insert (imaging artifact) and are not cellular nuclei. Scale bars, 50 µm. (**b**) Quantification of ZO-1 expression by mean fluorescence intensity (MFI) showing no significant difference between static and dynamic conditions (ns = not significant). (**c**) Transepithelial electrical resistance (TEER) measurements of Caco-2 monolayers under static (CO_ST) and dynamic (CO_DY) conditions. No significant differences were observed (ns). Data are presented as mean ± SEM from three independent biological replicates (*n* = 3).

To verify intestinal barrier integrity under dynamic co-culture, transepithelial electrical resistance (TEER) measurement and immunostaining of the tight-junction protein ZO-1 were performed, as these are the most reliable indicators of epithelial barrier function [24]. ZO-1 staining revealed continuous tight junctions under both CO_ST and CO_DY conditions (Figure 5a). Quantification of ZO-1 expression showed no significant differences between static and dynamic co-culture (Figure 5b). TEER values were consistently around 200 Ω·cm^2^ in both conditions, with no significant changes between CO_ST and CO_DY (Figure 5c). These results indicate that dynamic rocking preserved the epithelial barrier properties of Caco-2 monolayers.

## 4. Discussion

In this study, we developed and evaluated a 3D-printed, pump-free multi-organ-on-a-chip device that integrates intestinal, hepatic, and skeletal muscle tissues under gravity-driven oscillatory flow. By combining Transwell cell culture inserts for barrier and muscle compartments with hydrogel-embedded hepatocyte spheroids, this system recapitulates key aspects of inter-organ communication while maintaining accessibility and low operational complexity.

Our results demonstrate that dynamic perfusion enhances muscle maturation and metabolic activity, supports improved hepatic characteristics, and preserves intestinal barrier integrity. These results are consistent with previous reports showing that microfluidic perfusion promotes nutrient exchange and waste clearance, thereby enhancing cell functions [25,26]. In the muscle compartment, we used universal markers such as myosin heavy chain (MYH) and lactate secretion to evaluate maturation and metabolic function. MYH is a standard marker of myotube maturation [27], and lactate production reflects glycolytic metabolism in functional myotubes [28]. The increase in MYH expression and lactate levels under dynamic co-culture underscores the importance of mechanical cues and inter-organ communication. Although contractility measurements were not included, future studies will incorporate electrical stimulation to quantify muscle contraction within the device.

Albumin secretion is a key indicator of hepatocyte activity, and our findings demonstrate that both static co-culture and dynamic co-culture with intestinal and muscle compartments enhanced HepG2 expression of albumin compared with monoculture [29,30]. This improvement is consistent with the notion that hepatic cells depend on paracrine factors and metabolic inputs from other organs.

The preservation of tight junction protein ZO-1 and stable TEER values under dynamic culture indicates that intestinal epithelial integrity was not compromised by oscillatory flow. This is significant because barrier disruption often confounds co-culture systems, especially under mechanical stimulation. Our results suggest that the combination of Transwell cell culture inserts and gentle rocking provides a robust model for studying nutrient and xenobiotic absorption without compromising epithelial physiology.

Most existing multi-organ-on-a-chip platforms rely on complex microfluidic controllers or peristaltic pumps to achieve inter-organ communication. While powerful, such systems are often costly, technically demanding, and difficult to implement in non-specialist laboratories. In contrast, our 3D-printed device can be fabricated at low cost using widely available materials (ABS, PLA, TPU) and operated with a simple rocking platform. The modular design also enables rapid prototyping and adaptation to different organ configurations. These features address a major gap between cutting-edge OoC technologies and their practical adoption for routine use in drug testing, disease modeling, and academic research.

Despite the successful demonstration of a pump-free, 3D-printed multi-organ co-culture platform, several limitations should be acknowledged. First, the present study relied on immortalized cell lines (Caco-2 and HepG2), which provide reproducibility and ease of handling but do not fully capture the complexity and metabolic diversity of primary or iPSC-derived tissues. In particular, HepG2 cells have limited drug-metabolizing capacity, including low cytochrome P450 activity, and therefore, the hepatic readouts presented here (e.g., ALB expression) should be interpreted as indicators of hepatic phenotype/maintenance rather than as quantitative evidence of CYP-dependent drug metabolism. Second, the co-culture duration was limited to a short-term 3-day window, chosen to establish feasibility and ensure stable device operation. Longer-term experiments will be necessary to evaluate tissue survival, barrier stability, metabolic adaptation, and chronic responses. Third, although we confirmed basic functional markers such as ALB, MYH, and lactate-related metabolic activity, a detailed assessment of hepatic drug metabolism—including CYP activity, transporter-mediated kinetics, and metabolite profiling—was beyond the scope of this proof-of-concept work and will require extended culture periods and dedicated sampling strategies. Fourth, the current study did not include dynamic monoculture controls or systematic pairwise co-culture conditions under identical rocking settings; therefore, the relative contributions of mechanical stimulation (enhanced mass transport/low shear) versus inter-organ paracrine cross-talk cannot be fully separated in the present dataset.

Future studies will incorporate primary human cells or iPSC-derived lineages to enhance physiological relevance, together with quantitative biochemical assays and real-time biosensing modules (e.g., metabolite sensors, TEER monitoring, and contractility readouts). These advancements will open new avenues for studying metabolic disorders such as insulin resistance, sarcopenia, and non-alcoholic fatty liver disease. They will also enable more comprehensive pharmacokinetic and toxicological assessments—particularly when combined with metabolically competent hepatic models and CYP/transporter assays—ultimately strengthening the utility of this multi-organ platform for drug development applications.

## 5. Conclusions

In this study, we demonstrate that a low-cost, 3D-printed multi-organ-on-a-chip device can successfully support an intestine–liver–muscle co-culture under gravity-driven perfusion. Dynamic flow enhanced muscle differentiation and hepatic function without compromising intestinal barrier integrity, validating the system as a versatile and physiologically relevant platform. By bridging accessibility and functionality, this approach expands the potential of OoC technology for both basic research and translational applications in drug discovery and metabolic disease modeling.

## Data Availability

The datasets used and analyzed during the current study are available from the corresponding author on reasonable request.

## Supplementary Materials

The following supporting information can be downloaded at: https://www.mdpi.com/article/10.3390/mi17020180/s1, Figure S1: Design and flow characterization of the 3D-printed multi-organ-on-a-chip device; Code S1: Rocking-driven channel-flow and tracer transport simulation (Python); Video S1: Oscillatory fluorescein transport and mixing in the circulation channel under rocking.
